# Indocyanine Green Laser Angiography in the Setting of Tumescence

**Published:** 2019-01-07

**Authors:** Erica Y. Xue, Jerette J. Schultz, Paul J. Therattil, Jonathan D. Keith, Mark S. Granick

**Affiliations:** Division of Plastic and Reconstructive Surgery, Department of Surgery, Rutgers New Jersey Medical School, Newark

**Keywords:** angiography, epinephrine, indocyanine green, SPY, tumescent

## Abstract

**Objective:** Indocyanine green laser angiography utilizes fluorescent dye to assess tissue perfusion in real time. While various studies have recommended against the concurrent use of indocyanine green angiography with vasoconstrictors, few studies have described the intraoperative effect of tumescent wetting solutions on indocyanine green angiography and its subsequent impact on scan interpretation and clinical decision-making. **Methods:** A retrospective medical record review was performed for cases in which indocyanine green angiography was utilized to assess an area where epinephrine-based tumescent solution had been used. **Results:** The authors report 2 cases that utilized epinephrine-based tumescent solution along with intraoperative indocyanine green angiography assessment of the region. The first case was a bilateral reduction mammoplasty, whereas the second case was a nipple-sparing mastectomy with immediate tissue expander reconstruction. In both cases, intraoperative angiography predicted poor tissue perfusion while clinical examination showed good perfusion. Clinical examination was followed in both cases, with no additional tissue being removed. Neither case resulted in tissue necrosis despite angiography results. **Conclusions:** While indocyanine green angiography is a powerful tool in tissue perfusion assessment, there are some situations in which clinical examination must be used to determine tissue viability.

Indocyanine green (ICG) laser angiography is a vascular imaging methodology that has been used to visually assess superficial blood flow, allowing real-time assessments of skin perfusion to guide surgical decision-making.^1^ Factors associated with ischemia, such as the use of vasoconstrictors, may lead to reduced fluorescence during angiography assessment and longer wait times to fluorescence following ICG administration.^2^ Thus, intraoperative use of wetting solutions containing tumescent anesthesia may interfere with ICG angiography in assessing tissue perfusion. Here, the authors report 2 cases, a bilateral reduction mammoplasty and a nipple-sparing mastectomy with immediate tissue expander reconstruction, that both utilized ICG angiography after epinephrine-based tumescent solution was infiltration into the breast.

## METHODS

A retrospective medical record review of an inner-city practice population was performed. Inclusion criteria were cases that utilized ICG angiography to assess an area where epinephrine-based tumescent solution was used. No institutional board review approval was required because there was no access to identifiable patient information involved in the research process.

## RESULTS

**Figure 1:**
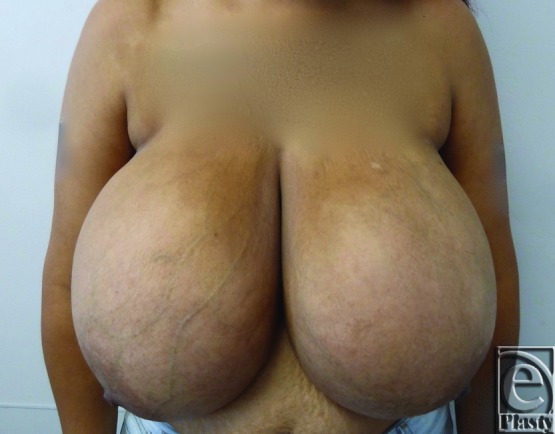
Case 1 preoperative photograph: A young woman with bilateral macromastia presenting for bilateral reduction mammoplasty.

Two patients were identified who met the criteria of this review. An otherwise healthy young adult woman with symptomatic bilateral macromastia presented for bilateral reduction mammoplasty (Fig 1). Preoperatively, the patient reported that she wore a size 48H brassiere. The patient underwent bilateral reduction mammaplasty with an inferior pedicle. Once the pedicle was de-epithelialized, wetting solution consisting of 1 ampule of epinephrine in 1 L of lactated Ringer's solution was infiltrated (450 mL of wetting solution on each side). In total, 1245 and 1605 g of tissues were removed from the right and left breasts, respectively. An additional 200 mL of fat was aspirated from the lateral and superior areas of the right breast using liposuction to optimize contour.

**Figure 2:**
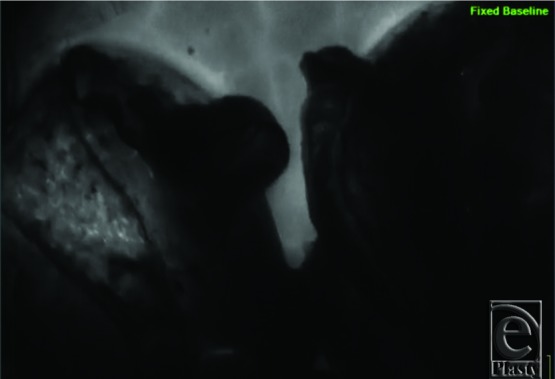
Case 1: Still photograph capture of SPY Elite indocyanine green angiography footage showing normal skin in the background with dye infiltration but minimal perfusion of the nipple-areolar complexes and breast pedicles.

**Figure 3:**
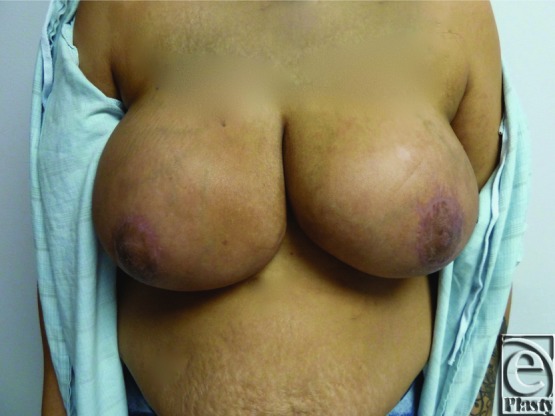
Case 1 postoperative photograph: Appropriate skin healing at 1 month.

Considering the size of the breasts and length of the pedicle, ICG angiography (SPY Elite Fluorescence Imaging System; NOVADAQ Technologies Inc, Richmond, British Columbia, Canada) was used to determine nipple viability. This was performed more than 90 minutes after the administration of tumescent solution. ICG angiography showed minimal perfusion of not only the nipple-areolar complexes (NACs) but also the entire breast pedicles, as well as the skin flaps (Fig 2). Despite this, clinical examination demonstrated viable NACs and skin flaps with good capillary refill and appropriate bleeding. As the ICG angiography results were presumed to be the result of tumescent solution use, the operation was completed without any additional excision of the tissue or free nipple grafting. The patient tolerated the procedure without complications and was discharged from the hospital on the same day. At 2- and 4-week follow-ups, the patient was healing appropriately with viable NACs and no areas of skin necrosis (Fig 3).

**Figure 4:**
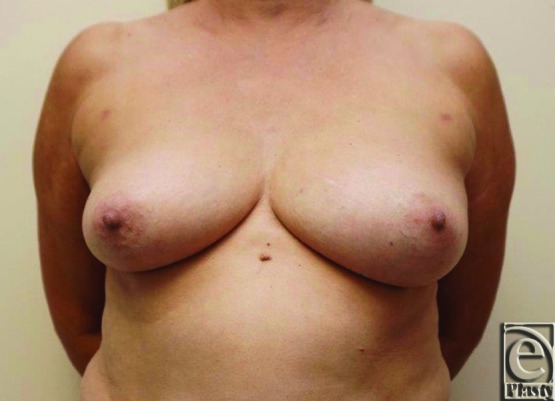
Case 2 preoperative photograph: A middle-aged woman with left breast cancer presenting for left nipple-sparing mastectomy and immediate reconstruction with tissue expander.

A middle-aged female with left breast cancer presented for left nipple-sparing mastectomy and immediate reconstruction with a tissue expander (Fig 4). The surgical oncology team performed the mastectomy, which took 45 minutes, using an inframammary approach. A 150 mL tumescent solution (1 ampule of epinephrine in 1 L of lactated Ringer's solution) was infused into the operative field prior to the initial incision.

**Figure 5:**
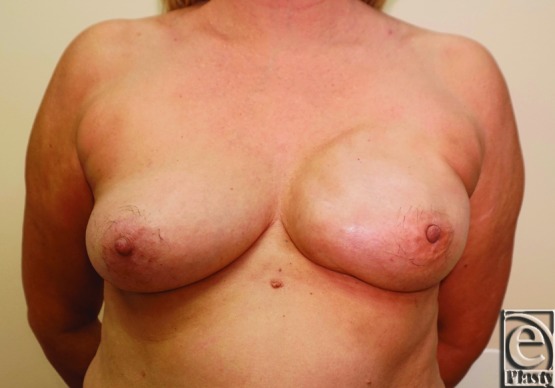
Case 2 postoperative photograph: Appropriate skin healing at 2 months.

SPY angiography was then used to evaluate perfusion of the mastectomy skin flap, which showed poor perfusion in the central portion surrounding and including the NAC (media 1). An additional 40 minutes elapsed during pocket preparation and tissue expander inset prior to reattempting ICG angiography. Repeat angiography revealed slightly improved uptake of ICG within the NAC and approximately 50% greater intensity of fluorescence. The poor perfusion on angiography was attributed to local tumescent effect and reconstruction proceeded. The patient tolerated the procedure without complications and was discharged from the hospital on the same day. Two months after the procedure, the breast had healed with a viable left NAC (Fig 5).

## DISCUSSION

Successful reconstruction is dependent on adequate tissue perfusion. While clinical judgment is essential in evaluating adequacy of blood supply, reliable technology now exist that can assess and quantify perfusion with a high degree of accuracy.^3^ ICG angiography is a popular choice for perfusion assessment due to its excellent safety profile and short plasma half-life that allows for repeat evaluations intraoperatively. The SPY Elite Fluorescence Imaging System utilizes ICG angiography and has been used to assess perfusion of free flaps, mastectomy skin flaps, and NACs during breast surgery.^2^

Vasoconstrictors, such as epinephrine, appear to preclude accurate ICG angiographic estimation of tissue perfusion due to temporarily diminished blood flow.^3^ Vasoconstriction following administration of epinephrine infiltration may necessitate longer wait times to ensure accurate ICG imaging.^2^ The SPY Elite Operator's Manual states that diminished blood flow following administration of epinephrine may indicate the need to wait 2 hours or more for accurate ICG imaging. A subsequent negative result on ICG angiography may indicate the need to wait even longer for recovery of perfusion. In the aforementioned cases, the authors waited 45 and 90 minutes, respectively, with some improvement in ICG uptake. It is unreasonable and impractical to prolong the patient's anesthesia time for the purpose of improved ICG uptake. These recommendations to avoid using ICG angiography following epinephrine administration do not specifically address the use of tumescent wetting solution containing extremely dilute amounts of epinephrine (on the scale of 1:1,000,000). The onset of activity for epinephrine takes up to 10 minutes, although its peak concentration in large-volume liposuction occurred at 1 to 4 hours after infiltration of the solution.^4^

**Video 1:** Indocyanine green angiography footage at 4× speed shows minimal perfusion of the mastectomy skin flap.

Tumescent solution, the combination of 1:1000 epinephrine with or without 0.5% lidocaine in 1 L of lactated Ringer's solution, was initially and most commonly utilized in liposuction surgery to minimize blood loss. Tumescent wetting solution has since been used to achieve hemostasis in other procedures such as mastectomies and reduction mammaplasties. Literature regarding the reliability of the ICG angiography in conjunction with tumescent solution is lacking—only one study has researched the efficacy of using ICG angiography to predict flap failure when epinephrine-containing tumescent solution was used. Munabi et al^3^ found that false-positive cases, defined as those with low perfusion values (≤7) that did not develop mastectomy flap necrosis, were more likely to have had an epinephrine-containing tumescent solution used during mastectomy. Thus, this study suggests that epinephrine-containing tumescent solution may yield low perfusion values that do not result in flap necrosis. This corresponds to the findings of low perfusion but subsequent benign clinical outcomes in both the aforementioned reported cases.

Our study is limited by the 2-patient case series design, which may not be generalizable due to the small number of subjects, has no control group, and is retrospective in its nature. More specifically, our study delves into the use of ICG angiography and tumescent solution in breast surgery. ICG angiography in the setting of tumescence may behave differently when applied to other tissues such as muscle and organs during other flap, gastrointestinal, or cardiovascular surgery.

## CONCLUSION

ICG angiography is a useful tool in assessing tissue perfusion, aiding surgical decisions regarding flap revision or resection of nonviable tissue, and predicting flap survival. However, the presence of even extremely dilute concentrations of epinephrine, such as that present in tumescent solution, can result in falsely low ICG infiltration and perfusion signals. Adequate time to degrade the epinephrine effect may lead to increased perfusion on repeat scan, although further studies are needed to assess the validity of this and to determine how much time is actually needed before accurate results can be obtained. The authors’ experience suggests that perfusion as assessed by ICG angiography in a field in which epinephrine-based tumescent solution has been used should be interpreted cautiously. When in doubt, clinical evaluation is the preferred assessment tool.
